# Supplementary material for: Systematics, Ecology, and Host Switching: Attributes Affecting Emergence of the Lassa Virus in Rodents across Western Africa
Source: Viruses. 2020 Mar 14;12(3):312. doi: 10.3390/v12030312 (PMC7150792; doi:10.3390/v12030312)
Supplement: Supplementary file 1 [file viruses-12-00312-s001.pdf]

viruses

MDPI

Supplementary Figure S1
Photos of rodent reservoirs of Lassa virus

Species	Country	Flanks	Belly
Mastomys natalensis	Guinea	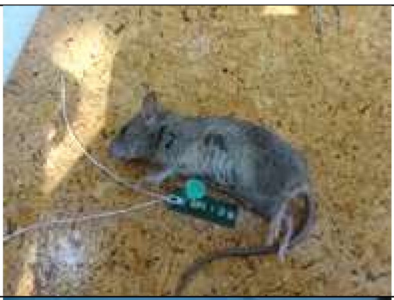	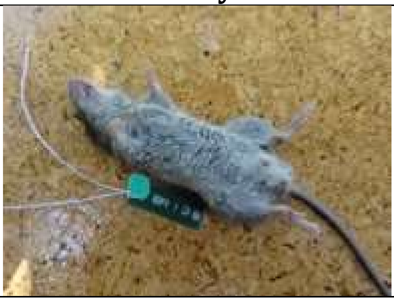
	Nigeria	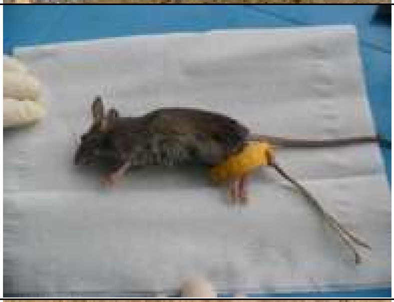	
Mastomys erythroleucus	Guinea	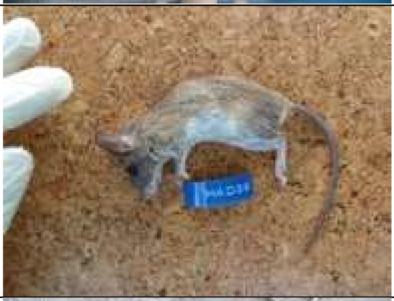	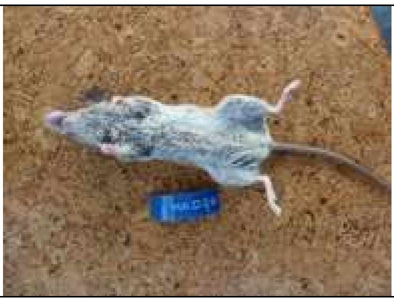
	Nigeria	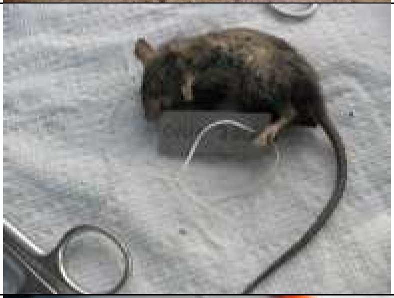	
Hylomyscus pamfi	Nigeria	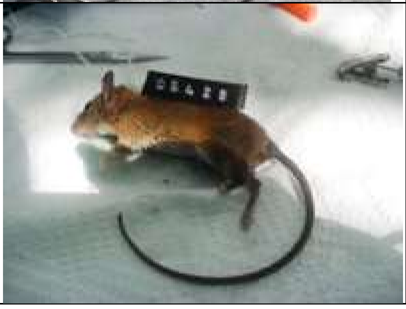	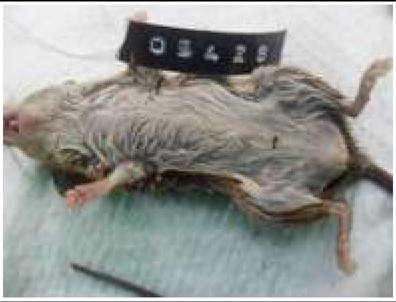

viruses

Mus baoulei	Nigeria	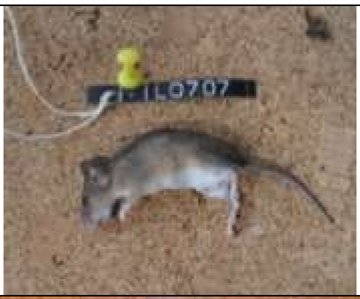	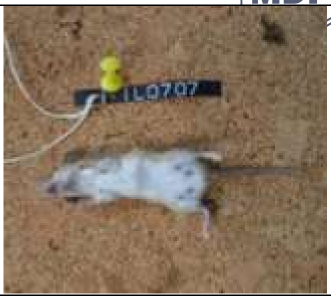
	Ghana	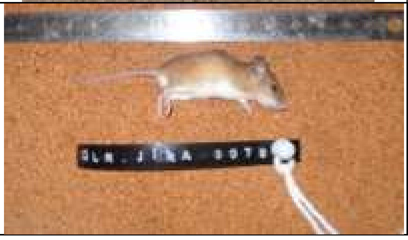	
